# Producers of Engineered Nanomaterials—What Motivates Company and Worker Participation in Biomonitoring Programs?

**DOI:** 10.3390/ijerph18083851

**Published:** 2021-04-07

**Authors:** Camille Crézé, Marjorie François, Nancy B. Hopf, Victor Dorribo, Jean-Jacques Sauvain, Enrico Bergamaschi, Giacomo Garzaro, Maida Domat, Judith Friesl, Eva Penssler, Athena Progiou, Irina Guseva Canu

**Affiliations:** 1 Department of Occupational and Environmental Health, Center for Primary Care and Public Health (Unisanté), University of Lausanne, 1066 Epalinges, Lausanne, Switzerland; marjorie.francois@unisante.ch (M.F.); nancy.hopf@unisante.ch (N.B.H.); victor.dorribo@unisante.ch (V.D.); jean-jacques.sauvain@unisante.ch (J.-J.S.); irina.guseva-canu@unisante.ch (I.G.C.); 2 Laboratory of Toxicology and Industrial Epidemiology, Department of Public Health and Pediatrics, University of Torino, 10126 Torino, Italy; enrico.bergamaschi@unito.it (E.B.); giacomo.garzaro@unito.it (G.G.); 3 Instituto Tecnológico del Embalaje, Transporte y Logística (ITENE), 46980 Paterna, Valencia, Spain; maida.domat@itene.com; 4 Yordas GmbH, 91301 Forchheim, Germany; j.friesl@yordasgroup.com (J.F.); e.penssler@yordasgroup.com (E.P.); 5 ALCON Consultant Engineers Ltd., 11257 Athens, Greece; ap@axonenviro.gr

**Keywords:** human biomonitoring programs, engineered nanomaterials, occupational exposure, exposure assessment, online survey, participation propensity

## Abstract

Production and handling of engineered nanomaterials (ENMs) can yield worker exposure to these materials with the potential for unforeseen negative health effects. Biomonitoring enables regular exposure and health assessment and an effective risk management. We aimed to identify factors influencing biomonitoring acceptance according to hierarchical positions of ENM producers. Managers and workers were invited to complete an online questionnaire. Forty-three companies producing or handling ENMs such as titanium dioxide (61%) and multi-walled carbon nanotubes (44%) participated. The majority of managers (72%) and all workers responded positively to participating in biomonitoring studies. The main reasons for refusing participation included concerns about data confidentiality and sufficient knowledge about ENM health and safety. Acquisitions of individual study results, improvement of workers’ safety, and help to the development of ENM-specific health and safety practice were among the most valuable reasons for positively considering participation. All workers indicated feeling comfortable with biomonitoring procedures of exhaled air sampling—about half were similarly comfortable with exhaled breath condensate, urine, and buccal cell sampling. The majority of both workers and managers stated that participation in a biomonitoring program should take place during working hours. Although our survey only had limited participation, our results are useful in designing appropriate biomonitoring programs for workers exposed to ENMs.

## 1. Introduction

Nanotechnology, i.e., the science and technology of matter on the nanometer scale, is a fast-growing industry that includes the production and handling of engineered nanomaterials (ENMs). Data from the 2012 Second Regulatory Review on Nanomaterials estimated that direct employment in nanotechnology amounted to 300,000–400,000 jobs in the European Union [1]. Their increased market-oriented production and handling can yield exposure with the potential for unforeseen negative health effects. In particular, workers in companies producing and handling nanomaterials are likely to have higher exposures to these materials than the general population.

Biomonitoring (BM) is an important tool to survey individuals’ internal exposure, i.e., the body burden of chemicals and/or their metabolites known as exposure biomarkers that accumulate from different sources of exposure, and the early biological effects known as effect biomarkers, highlighting, e.g., oxidative stress or inflammation [2,3]. The relevance of BM can be for the purpose of exposure assessment, risk management, and health surveillance [4,5]. Occupational BM programs are thus of particular interest during primary and secondary manufacturing of ENMs [6,7], i.e., processes identified as being at-risk of ENM exposure with unknown toxicity in humans [8,9,10,11,12,13,14]. Yet, despite the broad employment in the field of nanotechnology, biomonitoring programs for ENM-handling workers are limited in number and scope [11]. A systematic review identified seven BM programs measuring oxidative stress, inflammation, cardiovascular and genotoxicity biomarkers in the blood, urine, and exhaled-breath condensates (EBCs) of ENM-handling workers for the period 2000–2015; only one took place in Europe [15]. Reasons behind the scarcity of such programs include scientific, methodological, political, and regulatory challenges, as examined by Guseva Canu and colleagues, who concluded that lack of political and regulatory support are currently the most salient issues [16].

A recent questionnaire survey highlighted that a lack of legal enforcement, in addition to a lack of BM guidance values and limited toxicokinetic information, currently constituted the most cited obstacles for using BM data in risk assessment [3]. This survey targeted risk assessors, but not the issue of BM acceptance among managers and workers, and practical aspects affecting its feasibility. Addressing these issues is crucial when deciding how to best design a BM program in such occupational settings; ignoring it would result in failure during implementation due to insufficient adherence of participants, unexpected logistical constraints, and ineffective communication. Therein, our survey aimed at identifying factors influencing participation acceptance in a BM program in companies producing and/or handling ENMs. It was conducted in the framework of the European-funded NanoExplore project, which aims at building an integrated approach for exposure and health effect monitoring of engineered and incidental nanoparticles in various workplaces.

## 2. Materials and Methods

### 2.1. Survey Design

This survey was a cross-sectional study with two target populations: managers, including health and safety (H&S) specialists, and workers. As none of the previously conducted surveys has actually targeted workers [17,18,19,20], we designed our survey to capture both groups of participants within a company. Questionnaires were designed separately for managers and workers, i.e., both in terms of content and linguistic version.

### 2.2. Questionnaire Creation and Translation

We reviewed existing questionnaires used in previously published surveys and studies conducted in ENM-producing facilities to develop our questionnaires [17,18,19,20]. In particular, we also examined those developed within the French national program of epidemiological surveillance of ENM-handling workers (EpiNano) [21,22], the Canadian survey [19], and the survey led by the International Commission on Occupational Health (ICOH) [20], as they characterized ENM job exposures, the awareness of workers regarding ENM exposure and potential health effects, and exposure control measures implemented onsite. We also considered broader data on an individual’s motivations to participate in research programs in general [16,23,24]. Both questionnaires used in our survey were originally developed in English by the project team, which comprised 1 epidemiologist, 3 toxicologists, 2 occupational physicians, 3 occupational hygienists, 4 engineers, and 2 communication professionals. First, team members reviewed each question and response item for each questionnaire. Then, those accustomed in working with ENMs were asked to complete both questionnaires. All ambiguously formulated questions or response items were corrected before final approval. Both questionnaires were then transformed into electronic interactive versions subsequently tested by team members. Both manager’s and worker’s questionnaires are available in the Supplementary Materials.

#### 2.2.1. Questionnaire Aimed at Managers

The manager’s questionnaire comprised two parts. The first part included 18 structured questions focused on technical and H&S aspects of ENM-related processes. These included type of job activities involving ENMs, ENM type and physical form, number of employees handling ENMs, and existence of ENM-specific safety procedures and/or exposure monitoring program and control measures (availability of collective and/or personal protective equipment (PPE)). The second part was tailored to either higher-level managers (23 questions) or H&S specialists (15 questions), i.e., to account for differences in management responsibilities and scope. Questions were focused on reasons motivating company participation in research on ENMs, views on staff motivation, acceptability of exposure and BM procedures, and ability to accommodate research campaigns (time of year, duration, physical layout of the factories). The final part of the manager’s questionnaire included a request to disseminate by email the link to the workers’ questionnaire to their company workforce.

#### 2.2.2. Questionnaire Aimed at Workers

The worker’s questionnaire comprised 20 structured questions and was designed to understand workers’ individual motivations and their perceptions and feelings regarding BM. This questionnaire was translated into French, German, Greek, Italian and Spanish, and checked by native speakers before conversion into electronic interactive versions.

### 2.3. Survey Administration and Data Management

**Figure 1:**
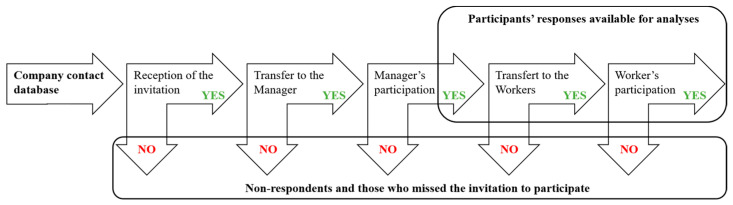
Dissemination strategy used in the framework of the NanoExplore survey.

The dissemination strategy is detailed in Figure 1. Electronic versions of the finalized questionnaires were uploaded on the Kwiksurvey platform (free online software provided by https://kwiksurveys.com/about, accessed on 8 April 2019). An invitation to participate in the survey was sent to 1774 company contacts previously assembled in a database by a project partner in the UK. This database contains contacts from nano-industry companies, research institutions, and other organizations involved in nanomaterial activities acquired through the partner’s participation in projects and events. The UK partner disseminated and managed the survey administration. The first email invitation was sent on 8 April 2019 and a reminder on 30 April. This resulted in 61 (3.43%) email delivery failures. In addition, an invitation to take part in the survey was posted on the NanoSafety Cluster (NSC) website and in the NSC May 2019 newsletter, in addition to regularly distributed project newsletters. The Nanotechnology Industries Association also disseminated our survey to their members. Other dissemination routes included social media, notably our UK partner’s and NanoExplore Twitter and LinkedIn accounts, and the NanoExplore project website. The survey was open from 8 April 2019 to 29 May 2020. To trace questionnaire dissemination steps, i.e., in particular from managers to workers, managers were asked to inform us on their decision to distribute the target questionnaire to their workers, and on how many workers received the link to the online questionnaire in their company. Data retrieval occurred through an automatically generated report that we analyzed in an Excel database in accordance with our research objectives. For each question, the absolute number of responsive companies and percent of the study sample are provided. Whenever relevant, responses are reported with indication of cut-offs imposed by the survey answer choices. The overall survey was managed in accordance with the European general data protection regulation.

## 3. Results

A total of 43 companies provided an answer to our survey, which represents a 2.42% response rate. Of these 43 companies, six declared not to be involved with any ENM-related activity. Nineteen companies provided an answer without completing the questionnaire, yet seven of them provided confirmation of ENM-related activity. Our analysis and discussion are based on the 18 completed managers’ questionnaires. We do not provide separate analysis by respondents’ position for this questionnaire (13 completed by managers and five by H&S specialists) because of the small number of respondents. Additionally, five workers completed the second questionnaire and qualitative results are reported below.

### 3.1. General Company Characteristics

Most of the respondent companies were located in Europe (*n* = 9; 51%), but some were located overseas (*n* = 4; 22%). The main activities involving ENMs were R&D activities (*n* = 12; 67%), nano-safety and industrial hygiene (*n* = 6; 35%), and production (*n* = 6; 35%). In half of the companies, the number of workers involved in ENM-related processes was 10 or less (*n* = 9; 50%), and three companies (17%) reported employing between 10 and 49 workers for ENM-related activities. Detailed values are reported in Table 1.

### 3.2. ENM Characteristics and Handling Specificities

**Figure 2:**
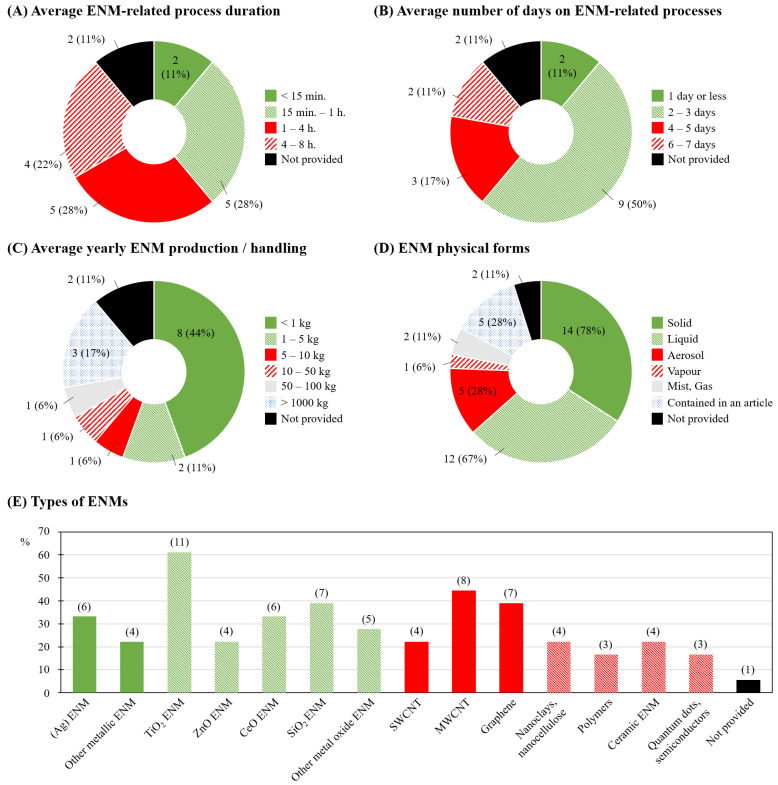
Engineered nanomaterials (ENM) characteristics and handling specificities: (**A**) average working process duration; (**B**) average number of days involving ENM-related working processes; (**C**) average yearly production/handling of ENM; (**D**) physical forms of ENM; (**E**) types of ENM manufactured/handled with number of respondent companies indicated in brackets. S/MW-CNT: single-/multi-walled carbon nanotubes.

The majority of companies reported having their employees work with ENM-related processes during 15 min to 1 h or 1 to 4 h per day (both *n* = 5; 28%; Figure 2A), for 2 or 3 days per week (*n* = 9; 50%; Figure 2B). Most companies indicated producing or using less than 1 kg of ENM per year (*n* = 8; 44%; Figure 2C), in majority in solid form (*n* = 14; 78%) or dispersed in a liquid (‘liquid’; *n* = 12; 67%; Figure 2D). The most commonly manufactured and/or handled ENMs were titanium dioxide (TiO_2_; *n* = 11; 61%), multi-walled carbon nanotubes (MWCNT; *n* = 8; 44%), and silicon dioxide (SiO_2_) and graphene (both *n* = 7; 39%; Figure 2E).

### 3.3. Health and Safety Plan and Practice

**Figure 3:**
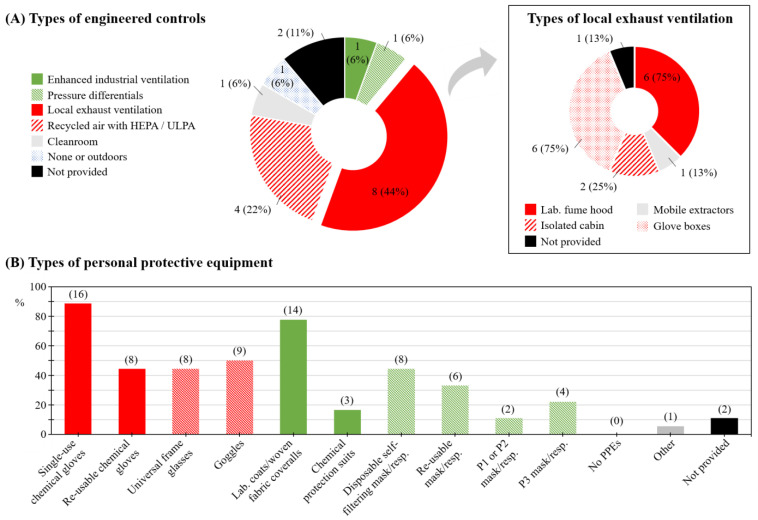
Health and safety (H&S) plan and practice: (**A**) Types of engineering controls. The square box on the right panel (*n* = 8) details types of local exhaust ventilation system; (**B**) Types of personal protective equipment (PPE) with numbers of respondent companies indicated in brackets. HEPA: high-efficiency particulate air filtration. ULPA: ultra-low particulate air filtration.

Thirteen companies (72%) reported having a nanomaterial-specific H&S plan (‘No’: *n* = 1; 6%; ‘Not provided’: *n* = 4; 22%), yet only two companies (11%) indicated following an ENM exposure monitoring program (‘No’: *n* = 12; 67%; ‘Not provided’: *n* = 4; 22%). Fifteen companies (83%) confirmed using engineering controls; local exhaust ventilation (LEV) and recycled air systems with high-efficiency particulate air (HEPA) or ultra-low particulate air (ULPA) being the most frequent (*n* = 8; 44% and *n* = 4; 22%, respectively; Figure 3A, left panel). LEV types were, in six out of eight (75%) companies, laboratory fume hoods and/or glove boxes (Figure 3A, right panel).

The use of PPE was widely reported. Single-use chemical protection gloves (*n* = 16; 89%), laboratory coats or woven fabric or cotton coveralls (*n* = 14; 78%), and goggles (*n* = 9; 50%) were the most frequently reported PPEs overall. Disposable self-filtering masks or respirators were reported by companies as the most frequently used type of respiratory PPEs (*n* = 8; 44%; Figure 3B). H&S specialists employed by the respondent companies were both occupational physicians and H&S specialists (engineer and technicians) (*n* = 6; 33%; and *n* = 10; 56%, respectively). Strategies used to manage the risk uncertainty related to ENM exposure were mostly based on an application of H&S procedures already in place for other substances (*n* = 5; 28%). Four companies (22%) reported reviewing the state of research regularly and updating their H&S procedures accordingly.

### 3.4. Managers’ Participation Acceptance and Study Practical Feasibility

**Figure 4:**
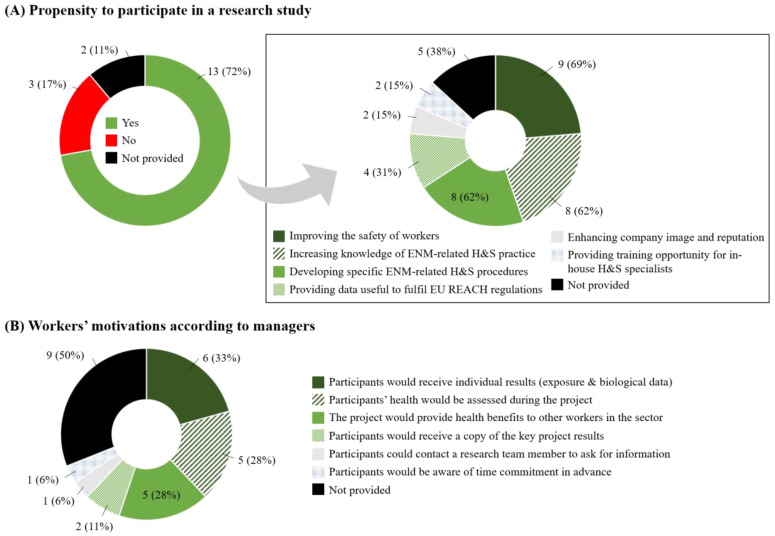
Managers’ propensity and reasons to consider participating in a research study. (**A**) Propensity to participate in a research study. The square box on the right panel (*n* = 13) details reasons for accepting participation. (**B**) Workers’ motivations to participate in a research study according to their managers.

A majority of the respondent companies (*n* = 15; 83%) had participated in a research study or a scientific partnership in the past (‘No’: *n* = 1; 6%; ‘Not provided’: *n* = 2; 11%). Thirteen companies (72%) confirmed that they would consider participating in a research study evaluating ENM exposure and possible impact on workers’ health, whereas three (17%) responded negatively (Figure 4A, left panel). Reasons for refusing participation were concerns about data protection and confidentiality (*n* = 1; 33%), or that in-house H&S specialists already had sufficient ENM information (*n* = 1; 33%). The main reasons for favorably considering participating in a research study were the improvement of workers’ workers (*n* = 9; 69%), an increased knowledge about ENM-related H&S practice, and help in developing specific ENM-related H&S procedures and practice (both *n* = 8; 62%). Four managers also indicated that such a study would provide useful data to fulfil obligations under the European Registration, Evaluation, Authorisation and Restriction of Chemicals (REACH) regulation [25] (Figure 4A, right panel). According to managers and H&S specialists, the main reasons for their workers to participate would be the acquisition of individual exposure and biological results (*n* = 6; 33%), assessment of their health during the project (*n* = 5; 28%) and indirect health benefits to other workers in the sector (*n* = 5; 28%; Figure 4B).

**Figure 5:**
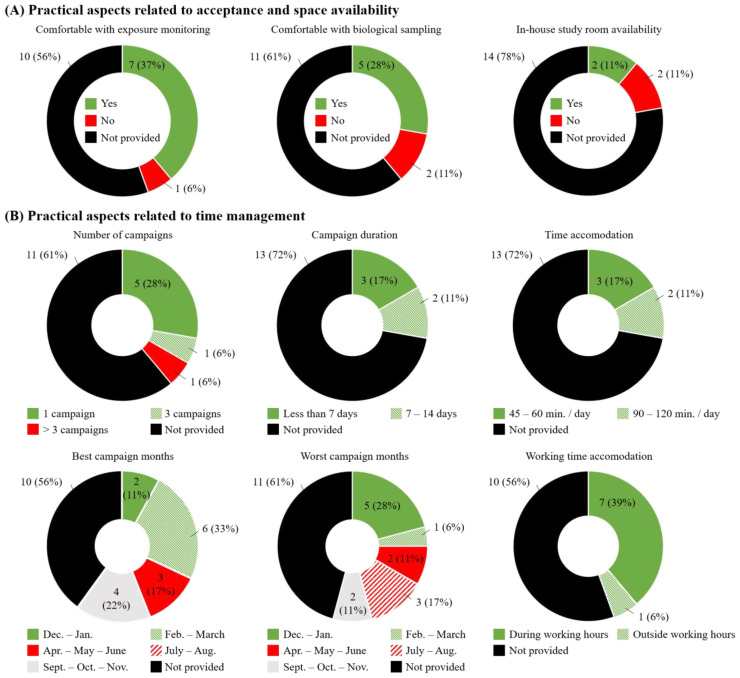
Managers’ responses regarding the feasibility of a research study and practical aspects associated with: (**A**) acceptance and space availability and (**B**) time management.

Regarding practical aspects of ENM research, several managers and H&S specialists indicated that they would be comfortable with ENM exposure measurements (*n* = 7; 37%), and sampling of biological media for their workers (*n* = 5; 28%; Figure 5A, left and middle panels, respectively). One of the practical hurdles of BM studies conducted in occupational settings is whether adequate space is available inside or near the working premises. This is specifically the case when EBC sampling is part of the study. Yet, only two companies (11%) confirmed the availability of an in-house room for biological sampling, and 14 of them (78%) did not know or did not provide a response (Figure 5A, right panel). Recognizing that participation in research programs can be time-consuming for companies, we also asked about expectations regarding acceptable duration and frequency of research campaign(s). Most managers reported preferring a study condensed in one campaign (*n* = 5; 28%) that would last 7 days maximum (*n* = 3; 17%), and require an hour per day or less of participants’ time (Figure 5B, upper panels). In general, managers reported spring and fall as being the most suitable periods to accommodate a research campaign (Feb.–March: *n* = 6; 33%; Apr.–May–June: *n* = 3; 17% and Sept.–Oct.–Nov.: *n* = 4; 22%; Figure 5B, lower left panel). Around the New Year period (Dec.–Jan.: *n* = 5; 28%) and summer months (July–Aug.: *n* = 3; 17%) were considered as the worst periods due to reporting workload and holiday time, respectively (Figure 5B, lower middle panel). All managers but one (*n* = 7; 39%) agreed on study procedures being conducted during employees’ working hours (Figure 5B, lower right panel).

### 3.5. Workers’ Participation Acceptance and Study Practical Feasibility

Five workers completed the worker’s questionnaire. Three workers from three companies reported being employed with a permanent contract and having worked for the company for 3 to 5 years, and the other two workers were either self-employed for more than 5 years or working under a short-term (3 months or less) contract. All workers confirmed that ENMs were produced and/or handled in the company, and that they handled ENMs at their workplace. Additionally, these workers indicated having received training regarding health effects and safety of ENMs.

These five workers unanimously reported willingness to participate in a study of ENM exposure and impact on their health. Furthermore, workers reported that acquisition of their individual results would be the main reason for participating, in accordance with what managers reported. Indirect benefits from such a study to the general population (including consumers) and to co-workers or other workers in the sector, and funding of the research study by a public institution, were also indicated as valuable reasons to consider participation.

All five workers confirmed that there was sufficient space for an ambient air-monitoring device at the workstation(s) where ENMs were produced and/or handled. Most workers confirmed feeling comfortable completing a questionnaire addressing their lifestyle habits and medical history, either administered face-to-face by a health professional or self-administered on a personal electronic device such as a tablet. When asked about biological sampling procedures, all workers reported feeling comfortable with exhaled air sampling; three of five workers were similarly comfortable with either one of the other procedures listed, i.e., exhaled breath condensate, urine, and buccal cell sampling. Workers unanimously stated that their participation in the research study should take place during working hours, in line with managers’ answers. Three workers considered allocating between 15 and 30 min of their time to a research study; the other two preferred either a shorter (15 min or less) or longer period (between 45 and 60 min). Three workers reported a preference for research procedures to take place only once per day, whereas the other two were ready to allocate their time to the research study as often as necessary.

## 4. Discussion

We identified several factors influencing participation for a BM program targeting ENM exposure and possible health effects in companies producing and/or handling ENMs. However, our findings are not generalizable, as the response rate was low, although similar to other surveys [20]. There could be several reasons for the low response rate. Because the dissemination strategy used in our survey included several intermediary electronic steps that could not be traced, calculation of exact response rates is difficult. Indeed, managers’ responses obtained here actually represent three steps: the invitation to participate was read by the email recipient, then transferred to the manager or H&S specialist, who read the invitation and agreed to participate. Some companies, in particular small companies and start-ups, might also have stopped their ENM-related business. As there is no unique, reliable source identifying such companies in the countries involved in this project, uncertainty remains regarding the number of existing companies actually producing and/or handling ENMs. Overall, 19 questionnaires of the 43 respondent companies were not able to be used. Furthermore, a number of managers or H&S specialists (*n* = 7) only opened the questionnaire to provide a confirmation of ENM-related activity; the survey was otherwise empty, thus highlighting the difficulty for researchers to trigger sufficient interest to recruit such companies. The situation with workers is even more complex, as it requests an effective transfer of the information from managers to their workforce, and the effective reception and treatment of this information by workers. Despite our request for managers to inform us if they disseminated the workers’ survey link to their workforce, none contacted us. Consequently, we could not consider the number of responses received from workers to calculate their response rate.

Insufficient interest and limited participation in research are common findings in previous studies conducted in ENM-producing and/or handling facilities. A low response rate was reported in a number of national and international surveys. For instance, the ICOH survey reported a 2.58% response rate despite extensive work to build a solid database of 2029 company contacts and the authority of the ICOH scientific committee “Nanotechnology workers”, which managed this survey [20]. Another survey conducted in 2012 in Quebec achieved a response rate of 8.4% from industrial companies contacted; however, this required considerable engagement from the study team as they individually called each of the 1181 companies in their database [19]. A Swiss survey conducted in 2007 provides the only contrasting example in the field [17]. Their response rate among companies was 58.3%, a figure mostly attributed to the fact that this survey was conducted jointly with the Ministry of Economy and Industry (SECO) and the national insurance provider (SUVA), which provides mandatory insurance, prevention services, and control of occupational H&S in Switzerland. This confirms an already expressed challenge to conduct health research and intervention without political and regulatory support [16].

Several lessons can be learnt from this survey. The first relates to the disparity between the number of responses received from managers and workers. The eight-fold lower number of responses from workers compared to managers attests to the challenges of reaching the ENM-handling worker population. Because no survey was previously conducted among workers, we examined the relative numbers of responses in the existing nationwide epidemiological program involving ENM-producing facilities. In a U.S. study of carbon nanotube and nanofiber exposure and health effects, conducted by National Institute for Occupational Safety and Health (NIOSH), the company participation rate was 18%, whereas the workers’ participation rate was 75% [26]. Similarly, in the French EpiNano program, the company participation rate was 16%, whereas the workers’ participation rate was 99% for a passive epidemiological follow-up and 42% for both passive and active follow-up [27]. This means that selection is significantly stronger at the company level than at the individual level. Our results confirm the self-selection at the company level but preclude discussion of workers’ selection. The challenge in accessing workers within this survey calls for a revised strategy allowing a facilitated or more direct access to the workers. For this, political and/or regulatory support would be necessary and can include ENM workers’ enrolment through worker unions outside companies.

Finally, our results suggest that respondent companies share a common characteristic not extensively described in the present survey. Indeed, most respondent companies reported having in place a specific H&S plan for working with ENMs, and a significant majority had already participated in other research studies or were involved with a scientific partnership, i.e., highlighting their common interest in and awareness of managing ENM-related issues. This common characteristic could manifest through more questions related to socially oriented traits, with an extensive investigation pertaining to awareness of the precise ENM exposure issue, or on how worker’s health and safety is perceived from the managers’ point of view. In light of the research scarcity in the field, it is particularly important that future studies address this self-selection issue.

The NanoExplore survey aimed to address the participation acceptance in a biomonitoring program targeting ENM exposure, and its practical feasibility in occupational settings among producers and handlers of ENMs. No published study previously addressed our research question, although the question of BM acceptance goes far beyond the field of ENMs. Considering the absolute number of responses and data provided in a field in which some aspects are still under investigation or unknown, our survey, although subject to a non-response concern, is helpful in designing communication strategies aimed at considering managers and workers’ expectations, in order to increase willingness to participate in occupational ENM exposure monitoring and biomonitoring programs. This survey captured a wide range of factors positively or negatively affecting company and individual engagement in ENM research, and provided valuable insights ranging from feelings and expectations regarding such programs to practical aspects regarding availability of space and time, or acceptability of planned BM procedures. Acquisition of individual study results, improvement of workers’ and the general population’s safety, and help in the development of ENM-specific H&S practices were among the most valuable reasons for positively considering participation. Results from this survey will inform further steps of the NanoExplore project (https://www.lifenanoexplore.eu/ (accessed on 8 April 2019)), which consists in designing a harmonized protocol for the BM of occupational ENM exposure and early health effects using biomarkers measured in non-invasive matrices. This survey is available in electronic or printed version, in six languages.

## Figures and Tables

**Table 1:** General characteristics of the 18 companies that participated in the study.

**Geographical Location**		**Number (%) of Companies**
Europe	Spain	3 (17%)
	Italy	2 (11%)
	Germany	2 (11%)
	Portugal	1 (6%)
	Norway	1 (6%)
Outside Europe	USA	2 (11%)
	Brazil	2 (11%)
Not provided		5 (28%)
**Sector for ENM-Related Activities**		
Production		6 (33%)
Use		4 (22%)
Storage, packaging, commercialization, distribution		3 (17%)
R&D, lab. use or characterization, scale-up		12 (67%)
Nano-safety, hygienist-related tasks		6 (33%)
Not provided		2 (11%)
**Number of Employees Manipulating ENMs**		
<10 employees		9 (50%)
10–49 employees		3 (17%)
50–250 employees		1 (6%)
>250 employees		3 (17%)
Not provided		2 (11%)

## Data Availability

Not applicable.

## Supplementary Materials

Managers’ and workers’ questionnaires (English versions) are available online at https://www.mdpi.com/article/10.3390/ijerph18083851/s1. File S1: NanoExplore Survey aimed at MANAGERS.
