# Supplementary material for: Producers of Engineered Nanomaterials—What Motivates Company and Worker Participation in Biomonitoring Programs?
Source: Int J Environ Res Public Health. 2021 Apr 7;18(8):3851. doi: 10.3390/ijerph18083851 (PMC8067629; doi:10.3390/ijerph18083851)
Supplement: Supplementary file 1 [file ijerph-18-03851-s001.zip › NanoExplore-Creze_MDPI-IJERPH_SupplMaterial.pdf]

<u>NanoExplore Survey aimed at MANAGERS – Part I</u>

Thank you very much for taking part in this short NanoExplore Project survey.

NanoExplore is a project mandated and funded by the European Community. It aims to evaluate health effects derived from exposure to engineered nanomaterials (ENMs) by setting up a harmonised health surveillance system, and promote new EU policies for the safe use of ENMs.

This short 10-minute survey will support us to identify the most commonly used engineered nanomaterials (ENMs) and provide information about exposure routes, release factors and the forms in which ENMs are placed on the market. It will also allow us to evaluate the factors and conditions that can facilitate or hinder the participation of companies in scientific research on nanomaterials.

This survey should be completed by a person with appropriate technical knowledge, this could either be a senior member of management or your company's health and safety manager.

If your company has multiple sites where engineered nanomaterials are produced or used, please fill in the information for the whole company (if you do know this information) or only for the site you manage or where you work. If providing information on one specific site, we would be grateful if you could then forward the link to this survey to the other sites' management team.

If you have any questions about this survey, please do not hesitate to contact the project team at info@lifenanoexplore.eu or at +44 (0)1524 510278.

IMPORTANT NOTICE: This is a voluntary survey. The information you provide will be kept confidential and used only for research purposes in accordance with the EU General Data Protection Regulation (https://eur-lex.europa.eu/legal-content/EN/TXT/?uri=celex%3A32016R0679).

1. **Before you proceed, please enter some basic information about your company**

Company's name
Country
Site location *(only if your company has multiple production sites and you will provide information only about that particular site)*

2. **Please also enter some information about yourself.** *This information is not mandatory but will be essential to provide you with feedback about the survey and its results. It will be kept confidential and only used for the purpose of this survey.*

First name
Last name
Email

3. **Does your company/institution produce or use engineered nanomaterials (ENMs)?**

A.Yes
B. No
If "B. No"

➔ *End of questionnaire text:*
"Thank you for participating in this questionnaire.
We are currently only looking for information from companies and institutions who work with ENMs.
If you know of anyone who works as a senior manager or a safety and health manager in a company/institution producing or using engineered nanomaterials, please forward them the link to this survey.
For any questions regarding this survey, please direct them to info@nanoexplore.eu."

4. **What exactly does your company/institution do with engineered nanomaterials (ENMs)?** *(Please select all that apply)*

A. Produce
B. Use (manufacturing articles or formulations/incorporate ENMs into products/disposal or recycling of products containing ENMs)
C. Storage/Packaging/Commercialization/Distribution
D. R&D activities/Lab use or characterization/Scale-up
E. Nanosafety/Hygienists related tasks
F. We do not use ENMs
G. I Don't know

5. **What is the physical form of the ENMs produced/used?** *(Please select all that apply)*

A. Solid (powder, granulates, etc…)
B. Liquid
C. Aerosol
D. Vapour
E. Mist / Gas
F. Contained in an article
Other (please specify)

6. **What is the approximate amount of ENMs that your company/institution produce or use annually?**

A. < 1kg/year
B. 1 kg/year up to < 5 kg/year
C. 5 kg/year up to < 10 kg/year
D. 10 kg/year up to < 50 kg/year
E. 50 kg/year up to < 100 kg/year
F. 100 kg/year up to < 1000 kg/year
G. 1000 kg/year and more

7. **How many employees handle ENMs in your company/institution?**

A. less than 10 employees
B. between 10 and 49 employees
C. between 50 and 250
D. more than 250 employees

8. **What is the average duration that an employee would work on a process where ENMs are produced or used on a typical day?**

A. less than 15 minutes
B. between 15 minutes and 1 h

C. between 1 and 4 h
D. between 4 and 8 h
Other (please specify)

9. **How many days on average would an employee work on a process where ENMs are produced or used on a typical week?** *(Sliding scale)*

0
1
2
3
4
5
6
7

10. **What are the different types of ENMs produced or used at your company/institution?** *(Please select all that apply)*

A. Silver nanomaterials
B. Titanium dioxide nanomaterials
C. Zinc oxide nanomaterials
D. Silicon dioxide nanomaterials
E. Cerium oxide nanomaterials
F. Zinc oxide nanomaterials
G. Graphene
H. Single-walled carbon nanotubes (SWCNT)
I. Multi-walled carbon nanotubes (MWCNT)
J. Gold and other metallic nanomaterials
K. Other metal oxide nanomaterials
L. Ceramic nanomaterials
M. Quantum dots and semiconductors
N. Dendrimers
O. Polymers
P. Nanoclays/nanocellulose
Q. I don't know
Other (please specify)

11. **Does your company/institution have a specific health and safety plan for working with nanomaterials?**

A. Yes
B. No
C. I don't know

12. **Which type of engineering controls are used to prevent exposure to ENMs?** *(Tick all that apply)*

A. General ventilation (1-3 air changes per hour)
B. Industrial ventilation (3-5 air changes per hour)
C. Enhanced industrial ventilation (5-10 air changes per hour)
D. Pressure differentials
E. Designed or separate working areas (e.g., control room)
F. Cleanroom
G. Local exhaust ventilation (fume hood, biosafety cabinet, glovebox, etc…)

H. Recycled air system with high-efficiency or ultra-low particulate air filtration (HEPA/ULPA)
I. None/outdoor
Other (please specify)

13. If you have answered "Local exhaust ventilation" in the last question, what types of local exhaust ventilation does your company/institution use?

A. Laboratory fume hood
B. Extractors incorporated in the tool
C. Mobile extractors
D. Isolated cabin
E. Glove box
Other (please specify)

14. What are the personal protection equipment (PPE) used by employees when handling ENMs? *(Please select all that apply)*

A. Chemical protection gloves (single-use) 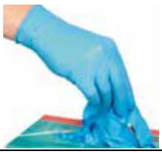	B. Chemical protection gloves (re-usable) 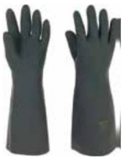
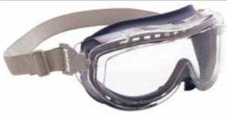 C. Goggles	D. Universal frame glasses 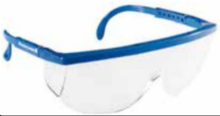
E. Laboratory coats/woven fabric or 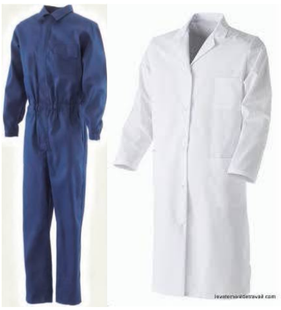 cotton coveralls	F. Chemical protection suit 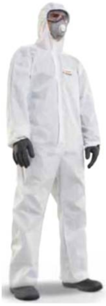
G. Disposable self-filtering 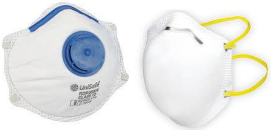 masks/respirators	H. Re-usable masks/respirators 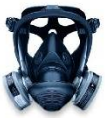 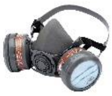
I. Masks/respirators are P1 or P2	J. Masks/respirators are P3
K. No personal protection equipment is used	
Other (please specify)	

15. What are the environmental protection measures used in your company/institution? *(Please select all that apply)*

A. Waste water is collected and treated at a waste water treatment plant (WWTP)
B. Vapours are collected and treated
C. Solid waste and used sacks/containers are disposed of according to local regulations

D. High efficiency particulate air (HEPA) or Ultra-low particulate air (ULPA) filtration in place with used filters disposed of according to local regulations
E. There are no environmental ENMs emissions to air, water, sediment and/or soil systems
F. I don´t know
Other (please specify)

16. **What kind of Health and Safety (H&S) specialist works in your company or institution?** *(Please select all that apply)*

A. Occupational physician
B. Industrial hygienist
C. H&S engineer
D. H&S technician
E. I don´t know

17. **Does your company/institution have an ENMs exposure monitoring programme in place?**

A. Yes
B. No
C. I don't know

18. **Which of the following best describes your job role in your company/institution?**

A. Managing Director / Senior Manager
B. Health and safety specialist

Depending on the answer to question 18, the respondent will access the relevant version of the second part of the questionnaire (one version is tailored to managing directors/senior management, the other is tailored to health and safety specialists)

NanoExplore Survey aimed at MANAGERS – Part II for MANAGING DIRECTOR / SENIOR MANAGER

19. **Has your company already participated in a research study or a scientific partnership?**

A. Yes
B. No
C. I don't know

20. **Would your company consider participating in a research study aimed at evaluating workers' potential exposure to engineered nanomaterials and possible impact on their health?**

A. Yes
B. No
If "A. Yes"
➔ *Question 21*
If "B. No"
➔ *Question 22*

21. **If you have answered 'yes' to the previous question, please indicate your reasons for doing so** *(Please select up to three reasons)*

A. Enhancing company image and reputation
B. Providing data that could be used to fulfil our regulatory obligations under EU REACH regulation
C. Improving the safety of workers
D. Providing training opportunity for our health and safety specialists/team
E. Increasing knowledge of health and safety practices related to engineered nanomaterials
F. Developing specific engineered nanomaterials health and safety procedures and practices
Other (please specify)

22. If you have answered 'no' to the previous question, please indicate your reasons for doing so *(Please select up to three reasons)*

A. Concerns about protection of proprietary information
B. Concerns about data protection and confidentiality
C. Concerns about company image or reputation
D. In-house health and safety specialists already have enough information about nanomaterials
E. Limited time available
F. Company already provides adequate protection to potentially exposed employees
G. Participation could raise concerns among employees regarding the safety of their working conditions
Other (please specify)

This type of research study usually comprises an exposure measurement and a personal biological sampling phase. During this phase of the study, the research team would come to the facility producing or using engineered nanomaterials and conduct these two study components (i.e. exposure measurement and biological sampling) simultaneously or separately; they could be repeated twice or more times over the project duration. Exposure measurements will not interfere with production or work processes. Some projects may last more than a year. The participating companies/institutions would receive a copy of the research study general results.

23. If your company/institution were to participate in such a research study, would it be comfortable with measurements of engineered nanomaterials exposure?

A. Yes
B. No
C. I don't know

24. If your company/institution were to participate in such a research study, would it be comfortable with bio-monitoring sampling and analysis in participating workers?

A. Yes
B. No
C. I don't know

25. What overall duration would your company be comfortable with for the exposure measurement and personal biological sampling phase? *(Please select applicable timeframe)*

A. Less than 7 days
B. 7 to 14 days

C. 14 to 21 days
D. 21 to 28 days
E. I don’t know

26. **How many measurement campaigns would your company be able to accommodate over the course of a year?** *(Please select all that apply)*

A. 1 campaign (all visits to the company site would be done on successive days)
B. 2 campaigns
C. 3 campaigns
D. More than three campaigns
E. I don’t know

27. **What would be the best period(s) for your company to participate in such a research study in 2020?**

A. January
B. February
C. March
D. April
E. May
F. June
G. July
H. August
I. September
J. October
K. November
L. December

28. **When would be the best period(s) for your company to participate in such a research study in 2021?**

A. January
B. February
C. March
D. April
E. May
F. June
G. July
H. August
I. September
J. October
K. November
L. December

29. **Please briefly explain why these particular times are most appropriate** *(Free text field)*

30. **When would be worst period(s) for your company to participate in such a research study in 2020?**

A. January
B. February
C. March
D. April

E. May
F. June
G. July
H. August
I. September
J. October
K. November
L. December

31. When would be worst period(s) for your company to participate in such a research study in 2021?

A. January
B. February
C. March
D. April
E. May
F. June
G. July
H. August
I. September
J. October
K. November
L. December

32. Please briefly explain why these particular times are least appropriate *(Free text field)*

33. Would your company be able to provide a temporarily dedicated facility/room for the research team to meet the study participants and perform biological sampling procedures and short interviews?

A. Yes
B. No
C. I don't know

34. Would your company prefer that employees participating in such a research study do so:

A. During their working hours
B. Outside of their working hours
If "A. During their working hours"
➔ *Question 35*
If "B. Outside of their working hours"
➔ *Question 36*

35. Would your company be able to accommodate the time needed to perform measures on each participating employee if the duration was the following *(Please tick all that apply)*

A. Between 45 and 60 minutes per day
B. Between 60 and 90 minutes per day
C. Between 90 minutes and 120 minutes per day
D. I don't know

36. If employee's participation to such a research study was to take place outside of their working hours, which of the following would you expect? *(Please select all that apply)*

A. Compensation would entirely come from the research study budget
B. Compensation would partially come from the research study budget
C. Your company would offer some kind of compensation
D. I don't know where compensation would come from

37. If able to provide compensation, in which form would the company's compensation be granted?

A. monetary compensation (overtime payment)
B. time-off in lieu
Other type of compensation (please specify)

38. According to you, what are the factors that would motivate your staff to participate? *(Please select up to three reasons)*

A. Assessment of their health at the beginning of and during the project
B. The project could provide health benefits to other workers in our sector
C. The project could provide health benefits to the individual participating
D. Participating employees would receive a copy of the study key results
E. Participating employees would receive individual results (exposure and biomonitoring data)
F. Participating employees could contact someone from the project team to ask for information
G. Participating employees would be aware of the time commitment in advance
Other (please specify)

39. Which strategies do you use to manage uncertainty regarding potential adverse health effects of engineered nanomaterials in your company? *(Please select all that apply)*

A. The level of uncertainty is of concern but information available is still very limited.
B. We are working with engineered nanomaterials that have been proven safe and we have no concern.
C. We are reviewing the state of research regularly and updating our health and safety procedures accordingly.
D. We are applying health and safety procedures already in place for other substances.
Other (please specify)

40. What kind of information would your regulatory affairs department need to facilitate your company's participation to such a research study? *(Free text field)*

41. Is there any information regarding engineered nanomaterials you wish you had? *(Free text field)*

Thank you for completing this survey.

As a complement to the questionnaire you have just filled, we are also interested in understanding how we can facilitate workers' participation in research projects on nanomaterials. To this end, we would kindly ask that you forward to your employees the link to the workers' survey (the survey is available in different languages).

To do so, you can easily copy and paste the link to the survey corresponding to the language of your country.

Please inform us of how many employees were sent the survey (info@nanoexplore.eu). It will allow us to determine the response rate.

Workers' survey: include survey link here
Sondage destiné aux travailleurs: include survey link here
Estudio per i lavoratori: include survey link here
Encuesta para trabajadores: include survey link here
Arbeiterumfrage: include survey link here
Έρευνα των εργαζομένων: include survey link here

NanoExplore Survey aimed at MANAGERS – Part II for H&S SPECIALISTS

19. Has your company already participated in a research study or a scientific partnership?

A. Yes
B. No
C. I don't know

20. Would you be interested in participating in a research study aimed at evaluating workers' exposure to engineered nanomaterials (ENMs) and possible impact on their health?

A. Yes
B. No
If "A. Yes"
➔ *Question 21*
If "B. No"
➔ *Question 22*

21. If you have answered 'yes' to the previous question, please indicate your reasons for doing so. *(Please select up to three reasons)*

A. Generating data useful for risk assessment (obligations under EU Reach regulation)
B. Identifying potential health and safety issues
C. Providing training opportunity for the health and safety specialists/team
D. Increasing my knowledge of health and safety practices related to engineered nanomaterials
E. Contributing to operational staff training on nanomaterials
F. Developing specific ENMs health and safety procedures and practices
Other (please specify)

22. If you have answered 'no' to the previous question, please indicate your reasons for doing so. *(Please select up to three reasons)*

A. Concerns about data protection and confidentiality
B. In-house health and safety specialists already have sufficient information about exposure to ENMs
C. The company health and safety specialist(s) have limited time and resources
D. The health and safety measures already in place provide adequate protection to potentially exposed employees

E. Participation may cause concerns among employees regarding safety of their working conditions
Other (please specify)

23. What would be the best period(s) for your company to participate in such a research study in 2020?

A. January
B. February
C. March
D. April
E. May
F. June
G. July
H. August
I. September
J. October
K. November
L. December

24. What would be the best period(s) for your company to participate in such a research study in 2021?

A. January
B. February
C. March
D. April
E. May
F. June
G. July
H. August
I. September
J. October
K. November
L. December

25. Please briefly explain why these particular times are most appropriate *(Free text field)*

26. What would be worst period(s) for your company to participate in such a research study in 2020?

A. January
B. February
C. March
D. April
E. May
F. June
G. July
H. August
I. September
J. October
K. November
L. December

27. **What would be worst period(s) for your company to participate in such a research study in 2021?**

A. January
B. February
C. March
D. April
E. May
F. June
G. July
H. August
I. September
J. October
K. November
L. December

28. **Please briefly explain why these particular times are least appropriate** *(Free text field)*

29. **According to you, what factors would motivate your company staff to participate in such a research study?** *(Please select up to three answers)*

A. General assessment of their health at the beginning and during the project
B. The project would provide health benefits to workers in our sector
C. The project would provide health benefits to the individual participating
D. Participating employees would receive a copy of the project key results (general results)
E. Participating employees would receive individual results (exposure and biomonitoring data)
F. Participating employees could contact someone from the project team to ask for information
G. Participating employees would be aware of the time commitment in advance
Other (please specify)

30. **According to you, what factors would make the management team consider company's participation to such a research study?** *(Please select up to three answers)*

A. Enhancing company image and reputation
B. Clear guarantee regarding protection of proprietary information
C. Clear guarantee regarding data protection and confidentiality
D. Fulfilling our regulatory obligations under EU REACH regulation
E. Time commitment known in advance
F. Participating companies would receive a copy of the project key results (general results)
Other (please specify)

31. **Which strategies do you use to manage uncertainty regarding potential adverse health effects of ENMs in your company?** *(Please select up to three answers)*

A. The level of uncertainty is of concern but information available is still very limited.
B. We are working with ENMs that have been proven safe and we have no concern.
C. We are reviewing the state of research regularly and updating our health and safety procedures accordingly.
D. We are applying health and safety procedures already in place for other substances.
E. We have developed internal regulatory values related to ENMs exposure
Other (please specify)

32. **Which kind of information would the health and safety department need to facilitate your company's participation?** *(Free text field)*

33. **Is there any information regarding engineered nanomaterials you wish you had?** *(Free text field)*

Thank you for taking the time to complete this survey.

As a complement to the questionnaire you have just filled, we are also interested in understanding how we can facilitate workers' participation in scientific study on nanomaterials. To this end, we would kindly ask that you forward to your employees the link to the workers' survey (the survey is available in different languages).

To do so, you can easily copy and paste the link to the survey corresponding to the language of your country.

Please inform us of how many employees were sent the survey (info@nanoexplore.eu). It will allow us to determine the response rate.

Workers' survey: include survey link here
Sondage destiné aux travailleurs: include survey link here
Estudio per i lavoratori: include survey link here
Encuesta para trabajadores: include survey link here
Arbeiterumfrage: include survey link here
Έρευνα των εργαζομένων: include survey link here

NanoExplore Survey aimed at WORKERS

Thank you very much for taking part in this short NanoExplore Project survey.

NanoExplore is a project mandated and funded by the European Community and conducted by a multidisciplinary project team involving university researchers. It aims to evaluate health effects derived from exposure to engineered nanomaterials (ENMs) by setting up a harmonised health surveillance system, and promote new EU policies for the safe use of ENMs.

This short 5-minute survey is an important component of the first phase of the project; the responses you provide will help us to understand how we can facilitate workers' participation in research studies aimed at monitoring workers' exposure to engineered nanomaterials and evaluating the potential effects on their health. During such a study, a research team would visit companies that have agreed to participate and identify work processes during which engineered nanomaterials are produced or used. Workers who are involved in these processes would then be asked if they want to participate in the study (participation would be strictly voluntary; workers can decide not to participate or leave the study at any point if they wish to do so). Following this preparatory phase, the research team would come to the company to measure whether engineered nanomaterials are released inside the working facilities and perform simple biological sampling procedures on participating employees. As part of these procedures, participants will be interviewed on some of their habits (for example smoking habits or medication use). All data obtained are strictly confidential and protected; the employee's company would have no access to them.

This is a voluntary survey.
The information you provide will be kept confidential and used only for research purposes in accordance with the EU General Data Protection Regulation (https://eur-

lex.europa.eu/legal-content/EN/TXT/?uri=celex%3A32016R0679). **It will not be shared with your company**.

Please read everything carefully and answer the questions as honestly as possible.

If you have any questions about this survey, please do not hesitate to contact the project team at info@lifenanoexplore.eu or at +44 (0)1524 510278.

In [please add relevant country], you can also contact [please add the name of a contact person that respondent can contact if they have any queries regarding the questionnaire or project] at [name of institution], at [insert email address] or at [insert phone number].

1. **Before you proceed, please enter some basic information about the company you work for:**

Company's name
Country
Site location *(if your company has multiple production sites)*

2. **If your company was to take part in a research study aimed at evaluating workers' potential exposure to engineered nanomaterials and possible impact on their health as described in the introductory paragraph to this survey, would you consider participating?**

A. Yes
B. No
If "A. Yes"
➔ *Question 3*
If "B. No"
➔ *Question 4*

3. **If you have answered 'yes' to the previous question, please indicate your reasons for doing so.** *(Please select up to three reasons)*

A. The project will contribute to protect my health.
B. Results will benefit my co-workers and generally, people working with nanomaterials.
C. Results will benefit the general population (as nanomaterials can be present in common products such as food items and cosmetics).
D. Participating workers will receive a copy of the general project results.
E. Participating workers will receive a copy of their personal data (exposure data and biological data)
F. The research study is funded by a public institution (For example the European Union, National health department/ministry)
G. The research study is managed by recognised university research centres
H. The results will be used primarily for public benefit.
Other (please specify)

4. **If you have answered 'no' to the previous question, please indicate your reasons for doing so.** *(Please select up to three reasons)*

A. Health and safety procedures and practices are strong in my workplace.
B. I am not worried about working with nanomaterials.
C. I am not comfortable with medical procedures.
D. I am concerned participation will take too much of my time.
E. I do not understand the objectives of the project.

Other (please specify)

5. **What is your age?**

A. 15-24 years old
B. 25-34 years old
C. 35-44 years old
D. 45-54 years old
E. 55-64 years old
F. 65 years and more

6. **How long have you worked for your current company?**

A. Less than 3 months
B. Between 3 months and 1 year
C. Between 1 and 3 years
D. Between 3 and 5 years
E. More than 5 years
F. Do not wish to respond

7. **What type of work contract do you have?**

A. Permanent contract
B. Fixed-term contract
C. Agency contract
D. Independent contractor (self-employed)
E. Other
F. Do not wish to respond

8. **Are engineered nanomaterials produced or used at the site where you are working?**

A. Yes
B. No
C. I do not know
If "B. No" or "C. I do not know":
➔ *Question 14*

9. **Are nanomaterials produced or used at any of your workstations?**

A. Yes
B. No
C. I do not know
If "B. No" or "C. I do not know":
➔ *Question 14*

10. **Have you received any health and safety training on nanomaterials?**

A. Yes
B. No

11. **At the workstation(s) where you work with nanomaterials, you working position is mainly:**

A. A seating position

B. A standing position
C. My position changes constantly
Other (please specify)

12. At the workstation(s) where you work with nanomaterials, is there a space where a small device (21 x 36 x 27 cm) can be placed so that it will not interfere with your work?

A. Yes
B. No
C. I do not know

13. If you consider the work you do with nanomaterials, how would you prefer to wear the small personal monitoring device that would be used (21 x 36 x 27 cm)?

A. Attached around the waist
B. Attached around the arm
C. In a small backpack
D. No preference

14. If you were to participate in a research study as described in the introductory paragraph to this survey, would you be comfortable with answering a study questionnaire? *This questionnaire would cover your life habits and medical history of importance in order to interpret the study results properly.*

- If the questionnaire were administered in face-to-face by a nurse/researcher: A. Yes / B. No
- If the questionnaire were self-administered using a pad/tablet: A. Yes / B. No
- If the questionnaire were self-administered using a printed form: A. Yes / B. No

15. If you were to participate in a research study as described in the introductory paragraph to this survey, would you be comfortable with the following simple biological sampling procedures in addition to a study questionnaire? *These procedures typically include non-invasive sampling of exhaled air (requiring quiet breathing using a disposable (non-reusable) device during 6 minutes maximum); exhaled breath condensate (requiring quiet breathing after applying a disposable nose clip for 15 minutes maximum); buccal/oral cells (requiring a soft brushing of mouth surface using a disposable brush during 5 minutes maximum), and urine (collected either in the morning, at home, or at the end of the working shift, using a sterile container).*

- Urine collection: A. Yes / B. No
- Exhaled air sampling: A. Yes / B. No
- Exhaled breath condensate sampling: A. Yes / B. No
- Oral cell sampling: A. Yes / B. No

16. What would be the maximum amount of time per day you would be willing to spend to undergo these biological sampling procedures?

A. Less than 15 minutes
B. 15 to 30 minutes
C. 30 to 45 minutes
D. 45 minutes to 1 hour

17. Would you prefer to have these procedures performed:

A. Once a day
B. Twice a day (pre- and post-shift)
C. As often as necessary
D. Other (please specify)

18. If you were to participate in such a study, the time commitment would be around one hour a day over the study period. Would you prefer for this time: *(Please tick one)*

A. To be taken on your usual working hours
B. To be taken outside of usual working hours
If "B. To be taken outside of usual working hours":
➔ *Question 19*

19. If your participation to the study was to take place outside of working hours, how would you prefer to be compensated for this time: *(Please tick one)*

A. Monetary payment (overtime payment)
B. Payment in kind (shopping vouchers, other…)
C. Time-off in lieu
Other (please specify)

20. Is there any information on nanomaterials you wish you had? *(Free text field)*

Before you leave this survey, please enter information about yourself.

This information is not mandatory but will be essential to provide you with feedback about the survey and its results. This information will be kept confidential and only used for the purpose of this survey.

First name:
Last name:
Email:
